# Visualizing neuroinflammation with fluorescence and luminescent lanthanide-based in situ hybridization

**DOI:** 10.1186/s12974-019-1451-2

**Published:** 2019-03-21

**Authors:** Lindsay M. Parker, Nima Sayyadi, Vasiliki Staikopoulos, Ashish Shrestha, Mark R. Hutchinson, Nicolle H. Packer

**Affiliations:** 1 Department of Molecular Sciences and ARC Centre of Excellence for Nanoscale Biophotonics, Macquarie University, North Ryde, NSW 2109 Australia; 2 ARC Centre of Excellence for Nanoscale Biophotonics, University of Adelaide, Adelaide, South Australia Australia; 3 School of Medicine, University of Adelaide, Adelaide, South Australia Australia; 4 Institute for Glycomics, Griffith University, Gold Coast, Queensland Australia

**Keywords:** Neuroinflammation, Time-gating microscopy, Neurokine, Microglia, Macrophages, Chronic pain

## Abstract

**Background:**

Neurokine signaling via the release of neurally active cytokines arises from glial reactivity and is mechanistically implicated in central nervous system (CNS) pathologies such as chronic pain, trauma, neurodegenerative diseases, and complex psychiatric illnesses. Despite significant advancements in the methodologies used to conjugate, incorporate, and visualize fluorescent molecules, imaging of rare yet high potency events within the CNS is restricted by the low signal to noise ratio experienced within the CNS. The brain and spinal cord have high cellular autofluorescence, making the imaging of critical neurokine signaling and permissive transcriptional cellular events unreliable and difficult in many cases.

**Methods:**

In this manuscript, we developed a method for background-free imaging of the transcriptional events that precede neurokine signaling using targeted mRNA transcripts labeled with luminescent lanthanide chelates and imaged via time-gated microscopy. To provide examples of the usefulness this method can offer to the field, the mRNA expression of toll-like receptor 4 (TLR4) was visualized with traditional fluorescent in situ hybridization (FISH) or luminescent lanthanide chelate-based in situ hybridization (LISH) in mouse BV2 microglia or J774 macrophage phenotype cells following lipopolysaccharide stimulation. TLR4 mRNA staining using LISH- and FISH-based methods was also visualized in fixed spinal cord tissues from BALB/c mice with a chronic constriction model of neuropathic pain or a surgical sham model in order to demonstrate the application of this new methodology in CNS tissue samples.

**Results:**

Significant increases in TLR4 mRNA expression and autofluorescence were visualized over time in mouse BV2 microglia or mouse J774 macrophage phenotype cells following lipopolysaccharide (LPS) stimulation. When imaged in a background-free environment with LISH-based detection and time-gated microscopy, increased TLR4 mRNA was observed in BV2 microglia cells 4 h following LPS stimulation, which returned to near baseline levels by 24 h. Background-free imaging of mouse spinal cord tissues with LISH-based detection and time-gated microscopy demonstrated a high degree of regional TLR4 mRNA expression in BALB/c mice with a chronic constriction model of neuropathic pain compared to the surgical sham model.

**Conclusions:**

Advantages offered by adopting this novel methodology for visualizing neurokine signaling with time-gated microscopy compared to traditional fluorescent microscopy are provided.

## Background

Neurokine signaling, which is measured by increases in the release of neurally active proinflammatory cytokines/chemokines and is indicated by regional low-grade glial reactivity, is a common pathophysiology in multiple central nervous system (CNS) disorders. Increased neurokine signaling arising from microglia and astrocytes occurs in response to injury and nociceptive events [1, 2], thus is prevalent in the establishment and maintenance of chronic pain in both animal models [3] and human patients [4]. Additionally, this low-grade glial reactivity and neurokine signaling is found in 40% or more of patients with bipolar disorder, schizophrenia, and obsessive-compulsive disorders [5] and is prominent in Parkinson’s [6] and Alzheimer’s diseases [7]. The development of new effective tools for visualizing and quantifying neurokine signaling is thus of high priority and interest to the neuroscience community. The detection of neurokine signaling molecules and their receptors can be challenging in vivo or in vitro [8].

Biomedical imaging is limited by the properties of established fluorescent molecules such as fluorescein, cyanine, and AlexaFluor dyes as they compete heavily with CNS tissue autofluorescence, which is especially high due to flavoprotein levels that coordinate with neuronal excitability and metabolic activity in vivo [9, 10], as well as reduced pyridine nucleotides (NADH, NADPH), and common neurotransmitter precursor amino acids such as tryptophan [11, 12]. Despite the competition with cellular and tissue autofluorescence, fluorescence imaging is the predominant method for imaging neurokine signaling, wherein fluorescently conjugated target molecules are detected by their absorption and emission characteristics.

The measurement of fluorescent decay (lifetimes) has proven that the dimension of time can also be used to detect optical signatures for dyes, autofluorescence, and nanoparticles [13]. Fluorescence lifetime imaging microscopy (FLIM) has been applied on light scattering brain tissues to study the dynamics of neuronal signaling activities with fluorophore-based fluorescence resonance energy transfer (FRET) imaging and in the intraoperative diagnosis of glioblastoma multiforme tumors via autofluorescence detection [14]. However, these and other studies in CNS tissues detect nanosecond range lifetimes that can have potential interference between the dyes and CNS cellular autofluorescence as their lifetimes can overlap.

**Fig. 1:**
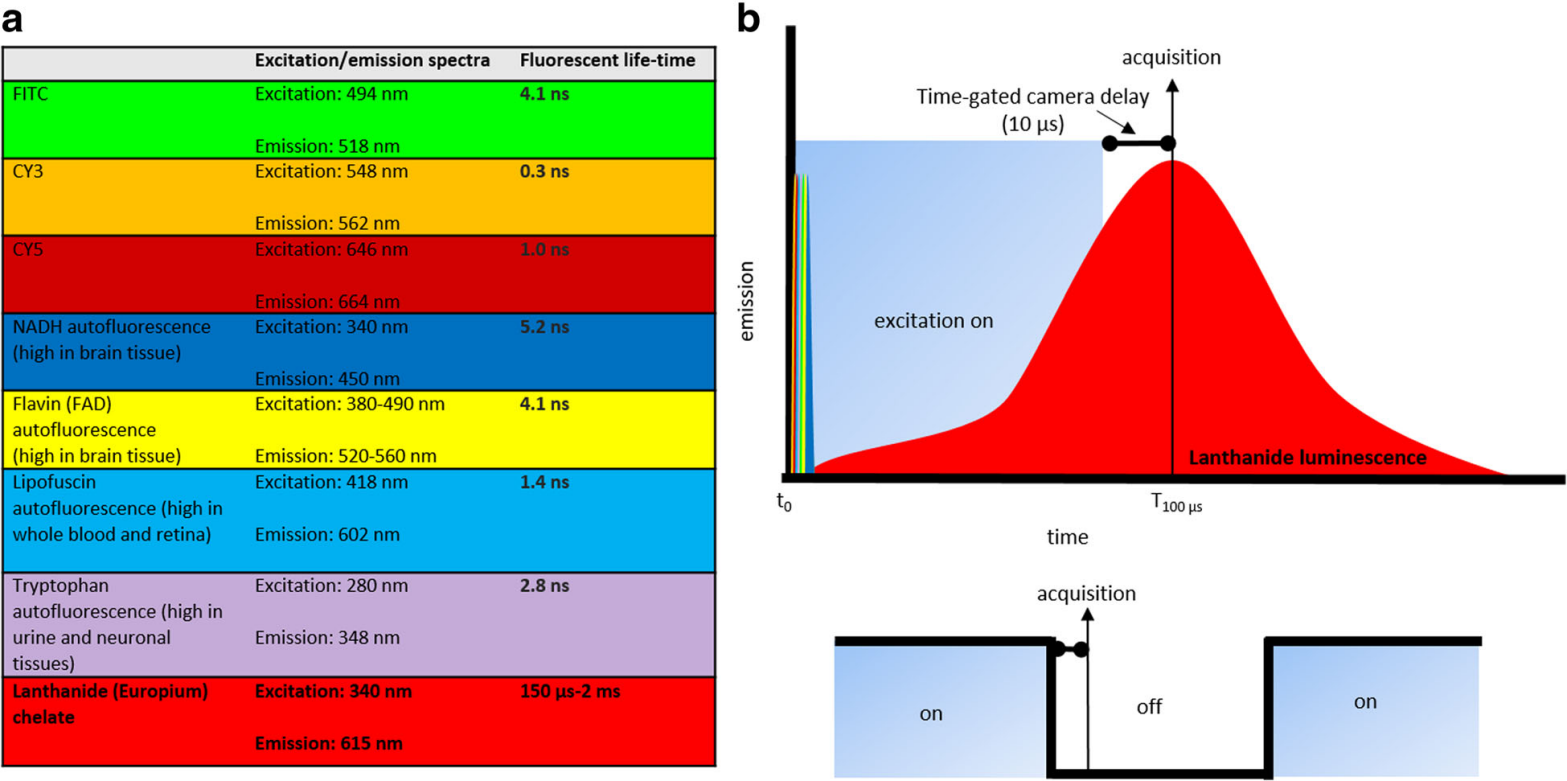
**a** Example fluorescent and luminescence lifetimes of common organic dye-derived fluorophores and autofluorescent molecules (denoted by corresponding color boxes/peaks in **a** and **b**) in comparison to europium-derived chelates (red in **a** and **b**). **b** The methodology of time-gated microscope excitation (light blue) and acquisition cycles. Colored peaks are representative of the relative short lifetimes of organic dyes and autofluorescence to long lifetime lanthanide luminescence. Light blue boxes are illustrative of the time of light excitation (excitation time per cycle = 100 μs), which is followed by a 10-μs period of delay before subsequent camera acquisition

Time-gated microscopy, whereby camera images are acquired at pre-determined delay times relative to the period of laser or wide-field light excitation (Fig. 1), is a promising technique that has previously been utilized for background-free imaging of proteins in microorganisms and cells [15–17]. Unlike traditional FLIM, time-gated microscopy better exploits the luminescence decay properties of luminophores that have longer lifetimes (up to 2 ms), providing contrast from autofluorescence that is unachievable by traditional fluorescence microscopy [7, 16, 18]. We have demonstrated that time-gated imaging combined with the use of a long-lifetime chemical probe, the recently developed biocompatible TEGylated europium chelate BHHTEGST (4,4′-bis-(1″,1″,1″,2″,2″,3″,3″-heptafluoro-4″,6″-hexanedion-6″-yl) sulfonylamino-tetraethyleneglycol- succinimidyl carbonate-*o*-terphenyl), can provide the opportunity to acquire background-free images with higher accuracy and confidence [18–21]. Unlike commonly used organic dyes, the luminescence lifetime of europium chelates is much longer than that of typical autofluorescence molecules (Fig. 1a, b), providing a greater signal detection sensitivity and discrimination against high levels of cellular background fluorescence for imaging neuroinflammation when combined with time-gating methodologies (Fig. 1b).

In order to demonstrate scenarios in which this methodology could be applied to benefit neuro-imaging, in this study, we have used cell models of immune activation and animal models of chronic pain to induce neurokine signaling and associated glial reactivity. Acute pain signals are meant to be adaptive and protective. They are communicated by well-established nociceptive neuronal pathways from peripheral sensory neurons to the dorsal horn of the spinal cord then onward to the brain through ascending fibers of the spinothalamic tract [22]. However, peripheral nerve injury can also lead to chronic pathological pain as continuous neurokine activity from glia and immune cells of the CNS can influence neuronal pain circuits for months or longer following injury in affected regions [23, 24]. The measurement of neurokine signaling has been primarily focused on detecting cell surface markers on activated microglia and/or the production of cytokine molecules. Identifying the response of other critical neurokine molecules such as the toll-like receptor 4 (TLR4) in glia and other immune cells that influence neuronal over-excitability helps to increase our understanding of the neuroimmune interface governing the pathophysiology of chronic pain and related diseases and disorders. The imaging of TLR4 is difficult due to the low protein expression levels of inflammatory mediator proteins [8] and the highly glycosylated surface of the TLR4 protein [25].

Biotinylated in situ hybridization (ISH) probes targeting mRNA coupled to streptavidin (SA)-conjugated luminescent lanthanides (LISH) or fluorescent Alexafluor dye were used in this study to characterize the presence of TLR4 mRNA. TLR4 is directly implicated in the development of and maintenance of chronic pain [26, 27]. It has been used as a marker of neuroinflammation following lipopolysaccharide (LPS) stimulation of microglia and was linked to macrophage survival in LPS-treated mice [28]. In order to provide examples of the usefulness of the new methodology we are presenting in this study in comparison to the current standard ways of visualizing mRNA transcripts, we visualized the expression of TLR4 mRNA in microglia and macrophage cells using the inflammatory response to LPS. We furthermore demonstrate TLR4 mRNA expression in the lumbar-level spinal cord using our methodology from mice that have symptoms of chronic pain. The current study has used luminescent europium chelate labels and time-gated microscopy to detect the RNA transcripts associated with inflammatory events in cells and tissue samples.

## Methods

### Cell culture and LPS stimulation

Mouse BV2 microglia phenotype cells and mouse J774 macrophage cells were grown in Dulbecco’s modified Eagle’s medium (DMEM) with high glucose (4500 mg/l), l-glutamine, sodium pyruvate, and sodium bicarbonate (D6429 Sigma) that was supplemented with 10% fetal bovine serum (12003C Sigma; Australian Origin), and 1% antibiotic-antimycotic (10,000 IU/ml penicillin, 10,000 μg/ml streptomycin, and 25 μg/ml of Fungizone® Antimycotic; 15240062 Life Technologies). The murine BV2 microglia cells were a generous gift from Prof. Gilles Guillemin (Macquarie University, Sydney, Australia), and J774 cells were a generous gift from Dr. Nisha Schwarz (SAHMRI, Adelaide, Australia). Cells were maintained in a humidified incubator with 95% air and a 5% CO_2_ atmosphere at 37 °C. All cells were sub-cultured for ISH experiments onto sterile, RNAse-free coverslips (22 mm × 22 mm) for 24–48 h inside six-well plates. The cells were incubated with LPS (0.5 μg/ml; tlrl-3pelps LPS-EB Ultrapure; InvivoGen) for the specified times under normal culture conditions.

### In situ hybridization probe design and synthesis

Primers for ISH were designed for this study using Primer3 (NCBI Nucleotide), spanning exon-exon junctions and including the protein-coding regions of the mouse TLR4 gene. The forward primer sequence includes the SP6 promoter sequence while the reverse primer sequence includes the T7 promoter sequence: TLR4 F primer with SP6 at 5′ end: 5′ATTTAGGTGACACTATAGAAG-TCCCTGCATAGAGGTAGTTCC 3′; R primer with T7 at 5′ end: TAATACGACTCACTATAGGGAGA-TCCCTGAAAGGCTTGGTCTT.

BV2 microglia cells were homogenized using the BeadBug Benchtop Homogenizer (Pathtech) and 0.5-mm beadblaster tubes with zirconium beads in lysis buffer containing 2-mercaptoethanol. Total RNA was then extracted using the PureLink® RNA Mini Kit (Life Technologies) according to the manufacturer’s instructions. The RNA was eluted from each column into 30 μl of RNase-free water. RNA quality and quantity were assessed using a Nanodrop 2000 spectrophotometer (NanoDrop Technologies, Wilmington, DE, USA). Reverse transcription of each RNA sample was performed using 1 μg of template RNA, 1 μl of 50 μM Oligo d(T)20 primer, and the Superscript IV reverse transcriptase (Life Technologies). Superscript IV First-Strand cDNA synthesis reaction: Oligo d(T)20 primer, 10 mM dNTP mix, template RNA, and water were mixed and denatured at 65 °C for 5 min, followed by cooling for 1 min on ice. 5× SSIV buffer, Superscript IV, 100 mM DTT, and RNAse inhibitor (RNAse Out) were added to the cooled template mixture and incubated at 55 °C for 10 min before enzyme inactivation at 80 °C for 10 min to make cDNA.

Standard PCR was performed for TLR4 using mouse BV2 cell cDNA. In each 25-μl reaction tube, there were 12.5 μl AmpliTaq Gold® 360 Master Mix (Life Technologies), 1 μl forward primer, 1 μl reverse primer, 1 μg cDNA, and 9.5 μl RNAse/DNAse-free water. Tubes were held at 95 °C for 10 min to activate the Taq enzyme followed by 40 cycle repeats of 95 °C for 30 s (denature), 60 °C for 30 s (anneal), 72 °C for 60 s (extend), and a final extension at 72 °C for 7 min. PCR products were purified using a column extraction kit according to the manufacturer’s protocol (PureLink® PCR Purification Kit; Life Technologies). PCR products were then run on a 2% TAE gel containing SYBR Gold and photographed on a Genesnap gel doc (Syngene) to confirm appropriate molecular weights and the absence of primer dimers. Purified PCR product was in vitro transcribed to complimentary RNA strands using the MEGAscript® T7 Transcription Kit (AM1334; Life Technologies) according to the manufacturer’s protocol using 200 ng of PCR product. Biotin-UTP and biotin-CTP were incorporated into the cRNA probe. RNA probe was purified with LiCl_2_ solution, washed with EtOH, air dried, and reconstituted in 40 μl of RNAse/DNAse-free water. RNA probe quality and quantity were then assessed using a Nanodrop 2000 spectrophotometer (NanoDrop Technologies, Wilmington, DE, USA).

### ISH for cellular microscopy

Cells were first washed in PBS pH 7.2 and fixed using 4% formaldehyde for 10 min and then again washed with PBS. They were permeabilized with 70% EtOH for 30 min at 4 °C then transferred to PBT solution (PBS + 0.01% Tween-20) until use. A subset of cells from each line (*n* = 6 coverslips per cell line) were pretreated before hybridization with an endogenous biotin blocking kit (E21390; Life Technologies). Pre-hybridization buffer (50% formamide, 5× SSC pH 7.0, 250 μg/ml herring sperm DNA, 5% dextran sulfate, 1× Denhardt’s solution, 0.1% Tween-20) was added to cells with 1000 ng of cRNA probe and incubated for ~ 16 h in an incubator at 37 °C. Cells were washed 3 × 5 min in 2× SSC buffer (sodium citrate/NaCl solution) followed by 3 × 5 min washes in PBT solution. Samples were incubated for 1 h at room temp (~ 22 °C) with streptavidin-Alexafluor555 (SA-555) or streptavidin conjugated-BHHTEGST Europium chelate (SA-Eu). Cells were then washed 3 × 5 min in PBT solution before being mounted onto slides in Prolong gold mounting medium containing DAPI (Molecular Probes) and photographed with wide-field microscopy (inverted Olympus IX83 or upright Olympus BX51). The BHHTEGST SA-Eu complex was produced as we have previously described [20], then lanthanide-based ISH was photographed with and without time-gating using a Gated Auto-synchronous Luminescence Detector (GALD) system [16, 18]. The FISH fluorescence was quantified by determining integrated densities, with the software ImageJ (National Institutes of Health, Bethesda, USA) using thresholding [29]. LISH fluorescence was measured using Zen Blue software (Zeiss) by spline contour tool and quantified by determining mean fluorescence for each group. Prism 7 (GraphPad Software, La Jolla, USA) or Excel was used for statistical analyses, and results were expressed as mean ± standard error of the mean (SEM). The *F* test of equality was used to test for normality of distribution in GraphPad Prism software (version 7.04) and revealed that there was significant variance in samples due to the presence of high and low responding cells following LPS treatment. Samples were therefore analyzed using the nonparametric Mann-Whitney test that does not assume a normal distribution, which in all cases revealed significance between groups to be *p* < 0.0001.

### Chronic pain “neuroinflammation” animal model

Pathogen-free adult male BALB/c mice (18–25 g, aged 8–10 weeks) were utilized for the experiments in this study. Mice were housed under SPF conditions in temperature (23 ± 3 °C) and light/dark cycle (12/12 h) controlled rooms with standard rodent food and water available ad libitum. Preceding experimentation, mice (*n* = 6 sham and *n* = 6 injury) were habituated to the animal holding care facility for 1 week, followed by 2 days of extensive experimenter handling and acclimatization to the von Frey testing apparatus in order to reduce successive handling stress. This study utilized an adapted version of the novel graded sciatic nerve injury model of allodynia [30], a modified CCI model based in the rat [31], where 0, 1, 2, 3, or 4 chromic gut sutures are placed around the sciatic nerve, to develop graded behavioral allodynia (varying degrees of allodynia) as described in detail previously [30]. To ensure the systemic chromic gut challenge was equivalent between animals [32], additional equivalent chromic gut lengths were placed subcutaneously over the hip, enabling each treated animal to be exposed to a total of four equivalent chromic gut pieces. Consequently, the injury and control treatment groups in this study only included six males in each treatment group. For this, the “injury group” received three chromic gut sutures around the sciatic nerve and one chromic gut suture subcutaneously, whereas the “control group” received no sutures around the sciatic nerve but four sutures subcutaneously. The mice were followed to postoperative (PO) day 21 to determine the extent of nerve injury.

The CCI model of sciatic nerve injury was performed at the mid-thigh level of the left hindleg as previously described [33]. Briefly, animals were anesthetized with isofluorane (2% in oxygen). The shaved skin was cleansed with 100% ethanol, and the surgery was aseptically performed. Zero (control group) or 3 (pain group) sterile chromic gut sutures (cuticular 4-0 chromic gut, FS-2; Ethicon, Somerville, NJ, USA) were loosely tied around the gently isolated sciatic nerve. Once the superficial muscle overlying the nerve was sutured, additional lengths of chromic gut were placed subcutaneously. Animals were monitored postoperatively until fully ambulatory prior to the return to their home cage and checked daily for any sign of infection. No such cases occurred in this study. Mechanical allodynia was assessed using the method described [30]*.* Briefly, mice were subjected to 10 stimulations with 6 calibrated von Frey filaments (0.04, 0.07, 0.16, 0.4, 0.6, and 1 g). The von Frey filaments were applied for 1 s at 1-s intervals. Filaments were not applied in ascending order of force, but rather random assignment each test session. In order to avoid sensitization, a break of 10 min was given between each set of stimulations, with 10 stimulations per filament. This method investigates the response frequency at each von Frey filament, and behavioral responses were recorded as the average number of responses out of 10 for each von Frey stimulus. The mice were tested for mechanical allodynia on the following days: pre-op day 1 and post-op days 1, 5, 7, 14, and 21. Two-way ANOVA showed that at day 21 there is an overall effect of surgery and hair weight on percentage mean paw withdrawal. A Tukey honest significance test was conducted with a 95% family-wise confidence level, the groups associated with each mean in the test were found to be normally distributed by residual, and Q-Q plots generated with R software (version 3.5.2).

### Tissue processing, ISH, and immunofluorescence

On day 21 of testing, the mice were perfused and then incubated with 4% paraformaldehyde (PFA) for standard fixation; *n* = 3 animals from each group were selected for ISH tissue processing. To prepare coronal slices for ISH, spinal cords were cut into 40-μm-thick free-floating sections on a cryostat. Finally, the prepared tissue slices were stained with ISH probes using the following protocol: tissue sections were incubated overnight in pre-hybridization buffer (50% formamide, 5× SSC pH 7.0, 250 μg/ml herring sperm DNA, 5% dextran sulfate, 1× Denhardt’s solution, 0.1% Tween-20) with 1000 ng of cRNA probe in an incubator at 58 °C. Sections were then washed 2 × 30 min in 2× SSC buffer (sodium citrate/NaCl solution with 0.1% Tween-20) followed by 2 × 30 min in 0.2× SSC buffer. Sections were washed 1 × 10 min in PBS + 0.1% Tween-20 solution (PBT). Samples were then incubated for 1 h at room temp (~ 22 °C) with streptavidin-Alexafluor488 (SA-488) or with streptavidin conjugated-BHHTEGST Europium chelate (SA-BHHTEGST-Eu^3+^). Sections were then washed 3 × 5 min in PBT solution before being mounted onto slides in Prolong Gold Antifade Mountant (P36930, Life Technologies) and photographed with wide-field microscopy at 10x magnification (upright Olympus BX51 microscope) with and without time-gating using a Gated Auto-synchronous Luminescence Detector (GALD) system [18]. SA-488 FISH was detected using 493-nm excitation and 517-nm emission, and LISH was detected at 340-nm excitation and 615-nm emission. All data are represented as mean ± SEM.

A proportion of spinal cord slices (*n* = 3 animals per group, *n* = 3 slices per animal) underwent immunofluorescence (IF) for glial fibrillary active protein (GFAP) to assess spinal cord astrocyte localization. Mouse spinal cords (L3) were fixed with 4% PFA, paraffin embedded and cut (5 μm) on a microtome. The tissue sections were hydrated and then antigen retrieval was carried out using citrate buffer (pH 6.0) for 10 min at 95 °C. Sections were blocked for 1 h at room temp with 10% normal donkey serum in 0.01% PBS-Triton (0.01 M) before adding mouse anti-GFAP conjugated to FITC (1:2000; eBiosciences cat# 53-9892) overnight at 4 °C. Sections were washed the next day using PBS (0.01 M) 3 × 10 min before adding DAPI nuclear stain for 5 min at room temp before washing once more with PBS (0.01 M) 3 × 10 min. Sections were cover-slipped using aqueous mounting media (Fluoro-Gel, Proscitech, IM030) and left to air dry. GFAP images were captured on a Leica SP5 Scanning Confocal microscope using a 490-nm excitation laser, and emission was detected at 525 nm using a 20x objective. For figures, images for FISH, LISH, and IF were adjusted equally for brightness and contrast using Inkscape software (version 0.91, www.inkscape.org) before arrangement into figures with Microsoft PowerPoint software.

### Lanthanide conjugation and detection

The BHHTEGST molecule contains an *N*-hydroxysuccinimide ester that enables it to be attached to streptavidin (CN: 43-4301, Life Technologies) via the amino group of lysine residues. For conjugation, 100 μg of streptavidin was exchanged into 100 mM NaHCO_3_ (pH 8.5) and then mixed with a 20-fold molar excess of the BHHTEGST ligand. After incubation for 1 h at 37 °C, the reaction mixtures were passed through a Sephadex G-25 column (2.0–2.5 g, 4.5-cm length, 0.9 cm ID) in 0.1× PBS (0.01% Tween 20, CAS Number 9005-64-5, Sigma Aldrich) to remove excess BHHTEGST. The fractions corresponding to labeled conjugates were collected according to absorbance detection using an Eppendorf BioPhotometer (280 nm and 320 nm). UV-visible absorption analysis of BHHTEGST (NanoDrop UV spectrometer) indicated a maximum UV absorption at 335 nm and also partial absorption at 280 nm which overlaps with that of the SA. To evaluate the partial absorption of BHHTEGST moiety in the conjugated SA, the molar extinction coefficient of the chelating tag at 335 nm and 280 nm were separately obtained from UV-visible analysis of purified BHHTEGST [ε_335_ = 3.14 × 10^4^ M^−1^ cm^−1^, ε_280_ = 1.75 × 10^4^ M^−1^ cm^−1^]. The concentration of the BHHTEGST was then obtained by reading the absorbance of conjugates at 335 nm (assuming that the extinction coefficient of BHHTEGST does not change on the labeled antibody). SA concentration was obtained by subtracting the absorbance of BHHTEGST from the absorbance of labeled protein at 280 nm. The number of BHHTEGST molecules per SA then was obtained by dividing the molar ratio of ligand to SA [20].

For the preparation of 10× fluorescence enhancing buffer (FEB) [34], the pH of a 44 ml solution of 0.1 M sodium hydroxide solution was adjusted to 4.7 with glacial acetic acid, then 1% by volume of Triton X-100 was added. Trioctylphosphine oxide (TOPO; MW 386, 38 mg) was dissolved in ethanol (5 ml) and added to the sodium acetate solution (1.25 ml). This solution was diluted to a working concentration of 1× FEB for all experiments. Coverslips with attached cells were placed upside down on microscopy slides on 5 μl of europium chloride [EuCl_3_, 20 mM in 1× FEB] and 20 μl of Prolong Gold anti-fade mounting medium with DAPI. Spinal cord slices were mounted in PBT buffer onto glass slides and cover slipped with 20 μl of Prolong Gold anti-fade mounting medium with DAPI and 10 μl of europium chloride. The labeled cells and spinal cord sections were examined using a DAPI filter on a conventional wide-field microscope (Olympus BX51 microscope) with and without time-gated luminescence mode using an opto-mechanical GALD device [35].

## Results

### Detection of TLR4 mRNA expression and autofluorescence in cells following LPS stimulation

**Fig. 2:**
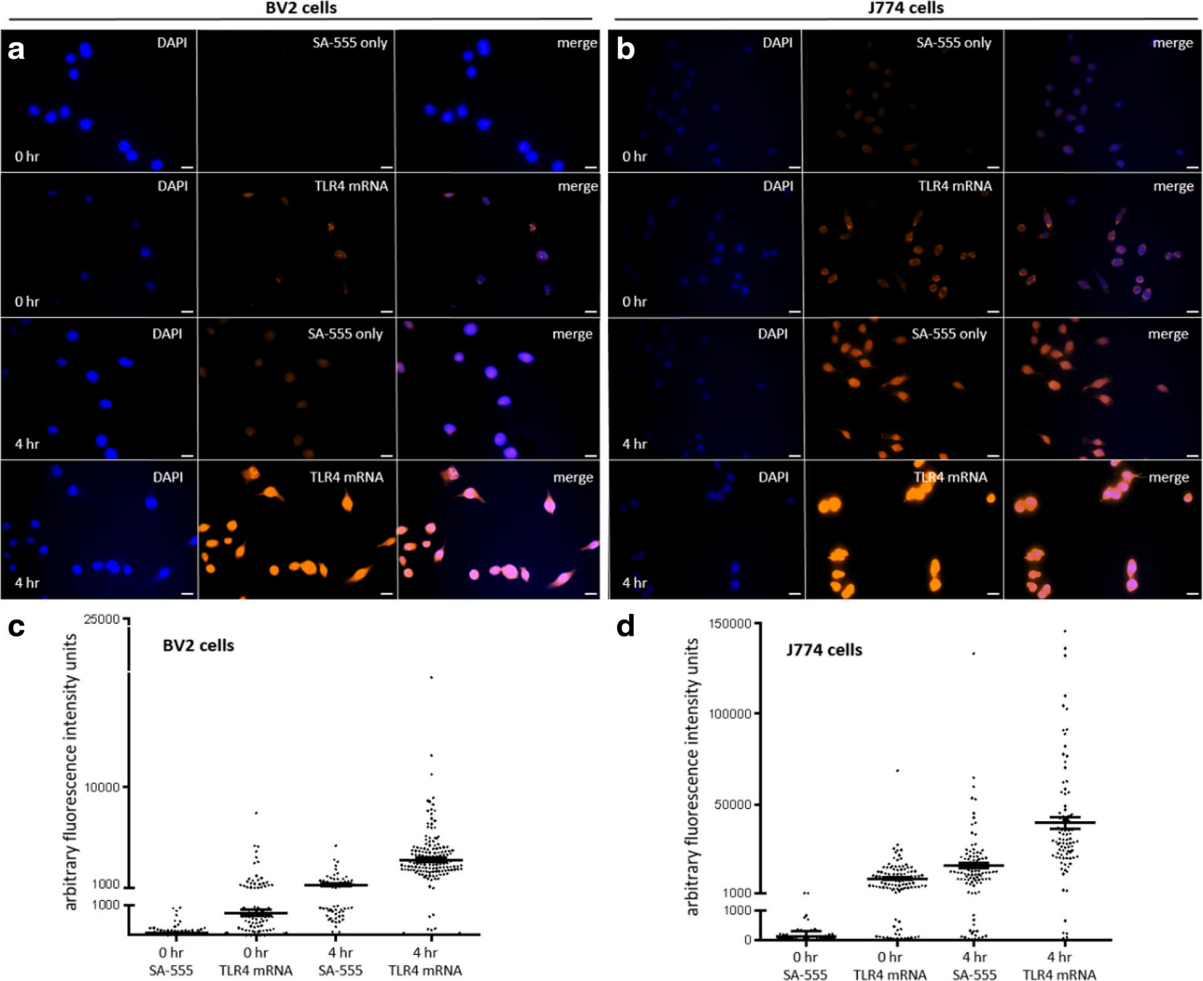
Toll-like receptor 4 (TLR4) mRNA staining in BV2 microglia cells and J774 macrophage cells (biotinylated TLR4 cRNA probe conjugated to streptavidin (SA)-Alexafluor555) following 0 h and 4 h of LPS stimulation (0.5 μg/ml) compared to cellular autofluorescence. **a** DAPI (blue) and BV2 cell autofluorescence or TLR4 mRNA expression (orange) following LPS-induced inflammation. **b** DAPI (blue) and J774 cell autofluorescence or TLR4 mRNA expression (orange) following LPS-induced inflammation. **c** Brightness intensities of LPS-treated BV2 cells represented in arbitrary fluorescence units. **d** Brightness intensities of LPS-treated J774 cells represented in arbitrary fluorescence units. 40x magnification, scale bar = 20 μm

Traditional fluorescent in situ hybridization (FISH) revealed that baseline TLR4 mRNA expression was low in most but not all BV2 cells (Fig. 2a). TLR4 mRNA expression in BV2 cells was significantly increased 4 h following LPS treatment with nearly all cells being highly labeled (*n* = 160 cells analyzed; Fig. 2c; *p* < 0.0001), and this was a 377% average increase in arbitrary fluorescence units compared to the 0 h treatment group (*n* = 114 cells analyzed). Cellular autofluorescence was also apparent in cells that were exposed to LPS for the same time periods but not exposed to any biotinylated TLR4 cRNA probe conjugated to SA-Alexafluor555. Autofluorescence in BV2 cells was significantly increased 4 h following LPS treatment (*n* = 64 cells analyzed; Fig. 2c; *p* < 0.0001) with an 898% average increase in arbitrary fluorescence units compared to the 0 h treatment group (*n* = 139 cells analyzed). Cellular fluorescence was however significantly higher in TLR4 mRNA-stained BV2 cells compared to autofluorescence at both 0 h (*p* < 0.0001) and 4 h (*p* < 0.0001).

J774 macrophage cells expressed significantly more TLR4 mRNA on average than BV2 cells at both 0 h and 4 h time points (*p* < 0.0001). TLR4 mRNA expression in J774 cells was significantly increased 4 h following LPS treatment with a range of expression intensities (*n* = 90 cells analyzed; Fig. 2d; *p* < 0.0001), and this was a 337% average increase in arbitrary fluorescence units compared to the 0 h treatment group (*n* = 115 cells analyzed). Baseline cellular autofluorescence was not significantly different between BV2 cells and J774 macrophage cells at 0 h; however, at 4 h following LPS, J774 cells had significantly more autofluorescence than BV2 cells at the same time point (*p* < 0.0001). As in BV2 microglia cells, autofluorescence in J774 macrophage cells was significantly increased at 4 h following LPS treatment (*n* = 105 cells analyzed; Fig. 2d; *p* < 0.0001) with a 13,789% average increase in arbitrary fluorescence units compared to the 0 h treatment group (*n* = 102 cells analyzed). However, cellular fluorescence of TLR4 mRNA-stained J774 cells was still significantly higher compared to autofluorescence at both 0 h (*p* < 0.0001) and 4 h (*p* < 0.0001).

**Fig. 3:**
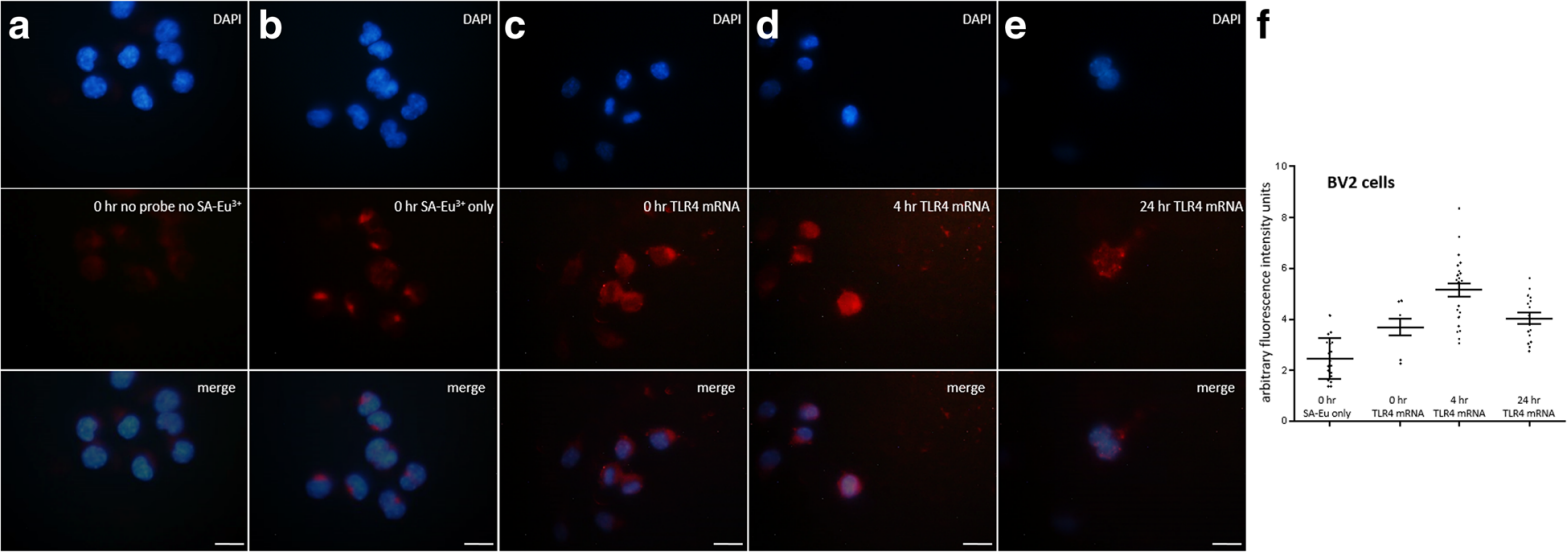
Luminescent lanthanide-based ISH staining in BV2 cells. **a** The detection of DAPI (blue; top panel) with epifluorescent microscopy and autofluorescence (red; bottom panel) with time-gated microscopy. **b** The detection of DAPI (blue; top panel) with epifluorescent microscopy and non-specific SA-Eu^3+^ binding (red; bottom panel) by time-gated microscopy. **c**–**e** The detection of DAPI (blue; top panel) with epifluorescent microscopy and the detection of TLR4 mRNA (red) by luminescent lanthanide staining and time-gated microscopy at **c** 0 h, **d** 4 h, and **e** 24 h of LPS-induced inflammation. **f** TLR4 mRNA was significantly increased from 0 to 4 h following LPS stimulation and subsequently returned to near baseline levels of expression by 24 h. A filter with 343-nm excitation/441-nm emission was used for epifluorescent microscopy (DAPI filter); 340-nm excitation/615-nm emission was used for SA-BHHTEGST-Eu^3+^ time-gated microscopy (100x magnification; scale bars = 100 μm)

Luminescent lanthanide chelate-based ISH (LISH) was also used for comparison to assess TLR4 mRNA expression in BV2 cells following LPS-induced stimulation. No background fluorescence was evident with time-gated microscopy when samples were tested in the absence of any probe or the absence of SA-BHHTEGST-Eu^3+^ (Fig. 3a). A small amount of staining was observed in cells exposed to SA-BHHTEGST-Eu^3+^ at 0 h but no RNA probe, suggesting a minimal level of endogenous binding (Fig. 3b). Low-level expression of TLR4 mRNA was observed at 0 h LPS stimulation (Fig. 3c–e), and these baseline levels were significantly higher than the RNA probe-free cells exposed to only the SA-conjugated lanthanide chelate and europium (Fig. 3f; *p* < 0.001; 49% average increase in arbitrary fluorescence units compared to the SA-BHHTEGST-Eu^3+^ only group). In agreement with our findings using FISH-based probes, TLR4 mRNA was increasingly expressed in BV2 microglia cells following LPS stimulation when detected by the biotinylated cRNA probe conjugated to SA-BHHTEGST-Eu^3+^ and time-gated microscopy; TLR4 mRNA significantly increased 4 h following LPS treatment (Fig. 3f; *p* < 0.006; 47% average increase in arbitrary fluorescence units compared to the 0 h treatment group). TLR4 mRNA levels were then subsequently significantly decreased to near baseline levels 24 h following LPS treatment (Fig. 3f; *p* < 0.005; 10% average increase in arbitrary fluorescence units compared to the 0 h treatment group).

### Measurement of the pain behavior response/glial reactivity in mice

**Fig. 4:**
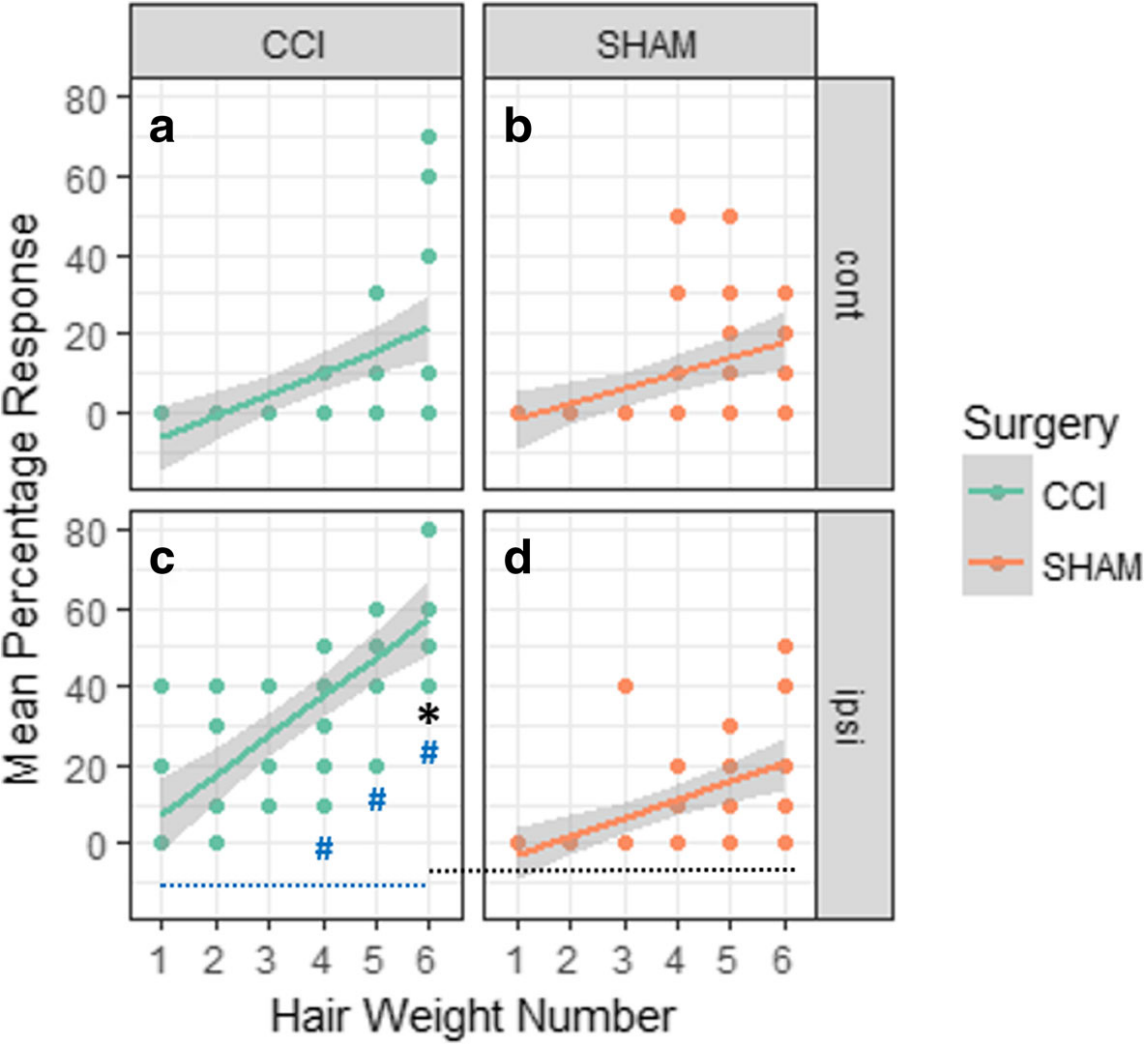
The mean percentage response of CCI-operated (green; *n* = 6) and sham-operated (orange; *n* = 6) mice on day 21 post-surgery (*p < 0.05) is shown for hair weights of 1 (0.04 g), 2 (0.07 g), 3 (0.16 g), 4 (0.4 g), 5 (0.6 g), and 6 (1.0 g). Contralateral hind paw shows no difference in mean percentage response between **a** CCI- and **b** sham-operated animals. Ipsilateral hind paw shows significant higher mean percentage response of CCI-operated animals in hair weight 1 g (**c**), compared to **d** sham-operated (*p < 0.05). Within CCI-treated animals, the mean percentage response was significantly higher in 0.4, 0.6, and 1 g hairs, compared to 0.04 g hair (^#^*p* < 0.05)

There was no difference in the mean percentage touch response observed in untreated contralateral hind paw of both sham and CCI (chronic constriction of the sciatic nerve) pain-induced mouse groups (Fig. 4a, b). At day 21, the treated ipsilateral hind paw of CCI mice showed a significantly greater mean percentage touch response to von Frey filament (1 g), compared to sham-operated mice (*p* < 0.05) (Fig. 4c, d).

### TLR4 mRNA detection in mouse spinal cord tissues

**Fig. 5:**
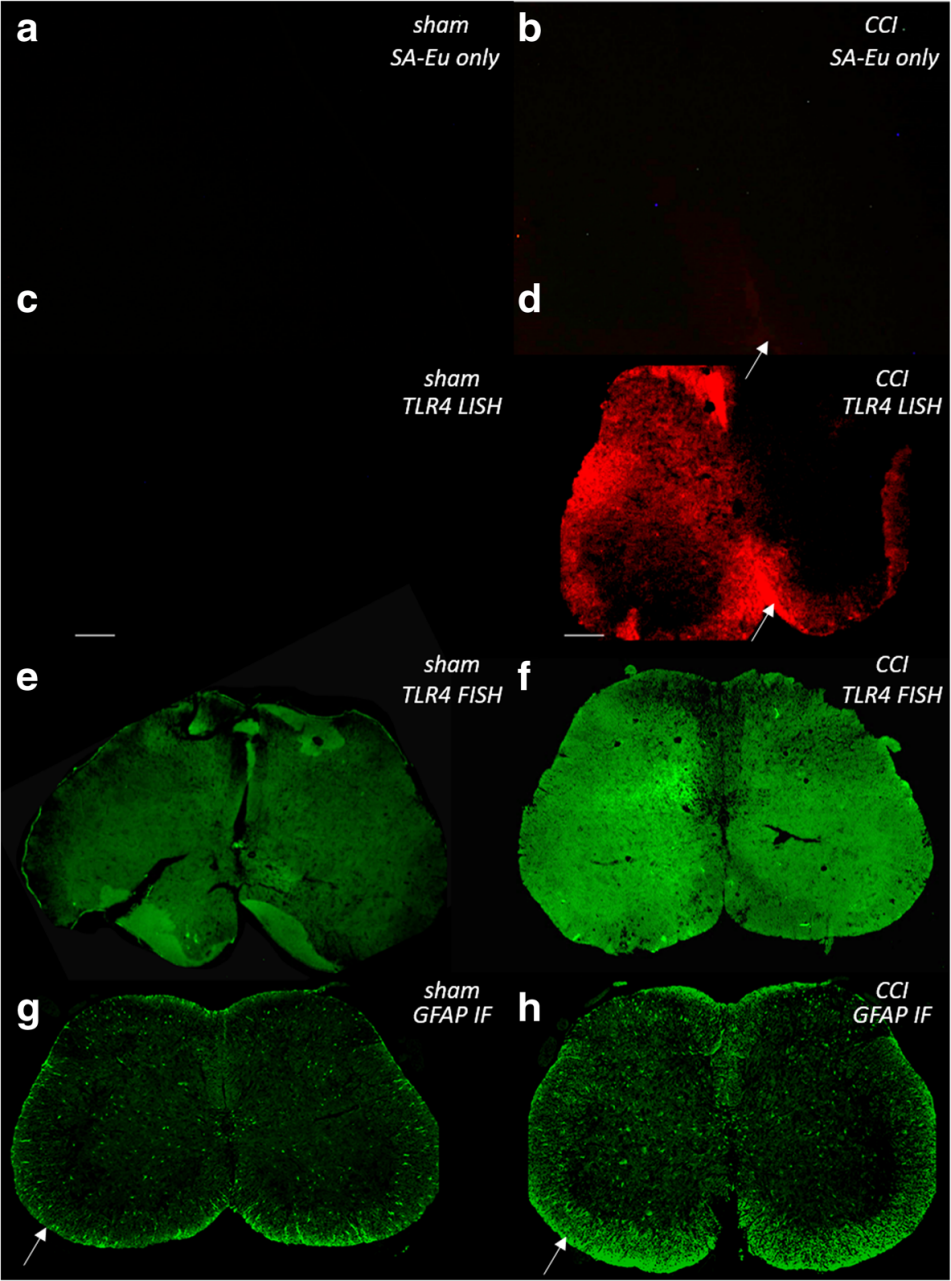
Fluorescence and luminescent lanthanide detection in L5 mouse spinal cords from sham-operated and CCI pain treatment mice are shown. **a**, **b** Sham and CCI pain animal spinal cord luminescence using only SA-BHHTEGST-Eu^3+^ chelate staining (**a**; without probe) and time-gated microscopy (**b**; weak area of SA-BHHTEGST-Eu^3+^ chelate background fluorescence indicated by arrow). **c**, **d** Sham or CCI pain animal spinal cord luminescence using LISH-based TLR4 mRNA staining and time-gated microscopy (**d**; strong region of TLR4 mRNA staining indicated by arrow). **e**, **f** Sham or CCI pain animal spinal cord luminescence using FISH (SA-Alexafluor488)-based TLR4 mRNA staining. **g**, **h** Sham or CCI pain animal spinal cord luminescence using GFAP immunofluorescence (IF), arrows indicate regions positive for GFAP protein. Scale bars = 100 μm

Little or no staining was observed with time-gated microscopy in the presence of SA-BHHTEGST-Eu^3+^ chelate alone in either surgical group (Fig. 5a, b). In the spinal cord collected from a sham-operated surgical mouse that did not experience mechanical allodynia pain, TLR4 mRNA staining with biotinylated in situ hybridization probes targeting mRNA coupled to streptavidin-conjugated luminescent lanthanides (LISH) was absent using time-gated microscopy (Fig. 5c). In contrast, LISH staining demonstrated noticeably strong TLR4 mRNA expression in the spinal cord of a mouse from the CCI pain group when viewed with time-gated microscopy (Fig. 5d). A predominantly unilateral increase in TLR4 mRNA was observed throughout the spinal cord in gray matter laminae I–VII and lamina X. Low levels of TLR4 mRNA expression on the contralateral side of the spinal cord may be due to the unilateral nature of the CCI pain induction model utilized, but this relationship was not directly assessed in the current study. The regions of the most intense signal were observed in the pyramidal tracts, axonal trafficking areas. TLR4 mRNA had a notably low expression throughout the ventral horn (Fig. 5d). Each CCI group animal (*n* = 3) showed some TLR4 mRNA staining localized to these regions, in two animals with high intensity, suggesting high levels of glial reactivity were present. Two animals from the sham-operated surgical group showed no LISH fluorescence with wide-field microscopy or time-gated microscopy, and one sham-operated mouse displayed a low level of TLR4 mRNA staining on the lateral and dorsal spinal cord edges indicating some glial reactivity could be due to the surgical process. Although the extent of TLR4 mRNA expression observed varied between mice from the CCI group, it was consistent within multiple L5 spinal cord slices from each animal (*n* = 3 slices). This suggests that there was a varying extent of glial reactivity and neurokine signaling in each animal.

For determination of detection sensitivity and competition with autofluorescence in tissue slices, TLR4 mRNA was also stained using FISH (tagged with SA-488) on additional sections from corresponding test mice. As expected, tissue autofluorescence was present in the mouse spinal cords in both treatment groups and competed with FISH detection of TLR4 mRNA (Fig. 5c—sham animal and Fig. 5d—CCI pain animal) making an accurate observation of regional TLR4 mRNA more difficult, particularly in the gray matter. In association with our TLR4 mRNA expression findings in the mouse spinal cord, we also observed a variable extent of astrocyte protein expression in mice from each group (*n* = 3 per group). Corresponding GFAP protein-stained sections taken from the same animals described above showed evidence of astrocyte-specific protein expressed in some but not all regions where TLR4 mRNA staining was observed (Fig. 5e—sham animal and Fig. 5f—CCI pain animal). This glial reactivity was highest in the ventral, dorsal, and lateral white matter spinal cord regions.

## Discussion

In the current study, we have highlighted two methods for visualizing molecular events associated with neurokine signaling in cells and tissue samples and have provided insight into dynamic TLR4 mRNA expression in microglia and macrophage cultured cells as well as in the spinal cord of an animal model of inflammation stimulated by pain. LISH-based mRNA staining results were comparable to using FISH for staining LPS-treated cultured microglial and macrophage cells. More dramatically, while FISH can be a valuable tool for measuring levels of neurokine signaling, long luminescent lifetime europium chelate conjugates visualized with time-gated microscopy (LISH) in neuronal tissue slices helped to abolish the competing fluorescent emission from tissue autofluorescence and enabled vastly superior resolution.

Lumbar level spinal cord autofluorescence is so apparent in rodent models of chronic pain that, in fact, autofluorescent flavoprotein imaging has been described as one direct method of visualizing regional increases in neuronal excitability and metabolic activity in vivo [9, 10]. The excitation and emission of endogenous flavoproteins occurs in the spectrum overlapping the most common commercial dyes excited by blue light (these include FITC, Alexafluor488, and Dylight488), therefore making nucleic acid probe and antibody targeting particularly challenging across the blue/green spectrum [11]. Additionally, unlike astrocytes, activated microglia and macrophages display substantial autofluorescent signals in rat brain in response to blue and green/yellow light excitation following neuronal injury due to an accumulation of lipofuscin granules [36]. Lipofuscin-derived autofluorescence is also apparent in activated microglia and infiltrating macrophages in the cortex of mice 3 days post treatment with i.p. LPS (5 mg/kg) [37], which may explain the noticeable increases in autofluorescence observed over time for the cellular models utilized in our study following LPS stimulation. We have presented evidence of competition with spinal cord tissue autofluorescence during blue light excitation when staining for TLR4 mRNA using a SA-488 tagged FISH probe. As demonstrated here for the detection of mRNA transcripts and previously to detect protein staining with an antibody, the use of long fluorescence lifetime europium chelates and time-gated microscopy provides the means to image cells and tissue slices with a background fluorescence-free environment. It is also possible to utilize other luminescent lanthanides such as terbium (Tb^3+^; λex = 350 nm, λem = 550 nm) as bio-probes with long lifetimes (> 100 μs) that are compatible with time-gated imaging [38, 39]. In this way, future studies could incorporate a multiplexed LISH approach for the simultaneous detection of more than one mRNA transcript.

RNA probes coupled to labeled molecules have contributed significantly to our understanding of RNA distribution throughout the brain and spinal cord and have identified neurochemical codes for some functional CNS cell subpopulations [40, 41]. They offer a valuable tool for detecting neurokine molecule expression and responses. This is particularly useful when protein identification is difficult due to poor antibodies and low protein copy numbers. Our findings agree with a previous report using real-time PCR in BV2 microglia cells that TLR4 mRNA is upregulated in a time-dependent manner following LPS stimulation, being highly significantly upregulated 4 h following LPS treatment [42]. In the current study, we were able to visualize these increases in TLR4 mRNA with fluorescent and luminescent lanthanide-based in situ hybridization and demonstrated that multiple cell phenotypes present in CNS tissue cross sections are capable of expressing TLR4 in response to inflammation. The comparability of our findings with different FISH conjugation methods and their agreement with previous other techniques quantifying TLR4 mRNA levels, gives us confidence in our lanthanide-based method for mRNA detection in cells, especially in the presence of high levels of autofluorescence such as in the CNS.

When using cRNA probes for the detection of mRNA in CNS tissues, it is common to use tyramide or RNAscope®-based signal amplification in order to help visualize fluorescent transcripts [43, 44]. We were able to demonstrate using luminescent lanthanide-labeled probes that signal amplification is not necessary if time-gated microscopy is used. Thus, these LISH-based probes provide greater fluorescent background suppression for CNS molecular imaging applications. Although the percentage increase in fluorescence detection was not as great for LISH in comparison to FISH, this is likely due to the drastically increased endogenous autofluorescence observed in the spinal cord tissue in this study. The autofluorescence may be associated with cellular mitochondrial energy demands and/or reactive oxygen species production, as has been seen in BV2 cells following LPS treatment [45]. There was a low level of background labeling in BV2 cells by the unconjugated (no probe) SA-europium chelate. We initially suspected that this labeling was due to the recognition of endogenous cellular biotin by the SA-europium chelate. However, we found that pre-treatment with an endogenous biotin blocking kit (Thermo Fisher Scientific #E21390) did not affect our results. Even though it has been reported that oligodendrocytes express higher levels of biotin for myelin production in neuronal tissues compared to other cell types, our spinal cord tissue samples showed little or no staining with SA-europium chelate alone. This suggests that the limited non-specific binding of SA-europium chelate is not correlated with endogenous biotin, but could be due to the minor hydrophobic/hydrophobic interaction of the europium chelate molecule to intracellular entities. We have similarly observed this low level of background labeling using the same europium chelate for lanthanide-based detection of DNA transcripts in *Staphylococcus aureus* (unpublished work).

TLR4-driven neuroinflammation is suspected to be involved in the initiation or maintenance of chronic pain symptoms in mice. For example, intrathecal application of LPS in mice induced increases in TLR4 mRNA levels (determined by real-time PCR) from lumbar levels 4–6 of male mice, and a pharmacological (LPS-RS) blockade of spinal TLR4 following spinal cord nerve injury in male mice drastically improved behaviorally assessed symptoms for pain [46]. Our current study provides an insight into the regional distribution of TLR4 mRNA expression and astrocyte localization in the CCI mouse model of chronic pain. Our findings are in agreement with another study demonstrating similar regional GFAP staining in the lumbar spinal cord white matter in other models of chronic pain in mice, where the authors interpreted their findings as a demonstration of substantial sustained astrogliosis [47].

As shown here, LPS stimulation resulted in increased expression of TLR4 mRNA in both microglia and macrophage cells. The TLR4 mRNA expression in the spinal cord tissues could potentially be multiple cell types within the regions. Microglia are known to express TLR4 whereas astrocytes and oligodendrocytes normally express little or no TLR4 mRNA [48, 49]. This suggests some of the observed mRNA expression is likely to be found within microglia cells in the tissue. The presence of microglia in the spinal cord white matter could be considered somewhat surprising, but reactive microglia have been seen in close apposition to axons in response to myelin reduction, which is a pathophysiology seen in the CCI model of murine chronic pain [50]. TLR4 mRNA has been demonstrated in murine macrophage cells from blood vessel tissues [51], and both macrophage and endothelial cells can bind LPS in mice following systemic administration [52]. There is additional evidence of macrophage cell involvement in neuroinflammation by their migration in response to released cytokines and macrophage responses to injury in spinal cord endothelial cells have also been reported [53].

## Conclusions

In summary, the methods demonstrated in this study can help neurobiologists to better visualize neurokine signaling (or other) molecules in cells or tissue samples. Using in situ hybridization can be a powerful tool for visualizing localized changes in mRNA expression of proteins that are hard to detect. We have also established that the use of luminescent lanthanide chelates visualized by time-gated microscopy can decrease imaging artifacts by allowing detection of the luminescent mRNA probe in the presence of the well-described increases in autofluorescence seen in inflamed cells and tissues.

## Abbreviations

BHHTEGST: 4,4′-Bis-(1″,1″,1″,2″,2″,3″,3″-heptafluoro-4″,6″-hexanedion-6″-yl) sulfonylamino-tetraethyleneglycol-succinimidyl carbonate-*o*-terphenyl
CNS: Central nervous system
Eu: Europium
FEB: Fluorescence enhancing buffer
FISH: Fluorescent in situ hybridization
FLIM: Fluorescence lifetime imaging microscopy
FRET: Fluorescence resonance energy transfer
GALD: Gated Auto-synchronous Luminescence Detection
GFAP: Glial fibrillary acidic protein
ISH: In situ hybridization
LISH: Luminescent in situ hybridization
LPS: Lipopolysaccharide
NADH: Nicotinamide adenine dinucleotide
NADPH: Nicotinamide adenine dinucleotide phosphate
SA: Streptavidin
TLR4: Toll-like receptor 4
